# Impact of the COVID‐19 Pandemic on Cancer Management in Iran: A Review

**DOI:** 10.1002/hsr2.72299

**Published:** 2026-04-05

**Authors:** Sepideh Hajivalizadeh, Mohammad Javad Mansourzadeh, Arash Ghazbani, Elham Ehsani, Ali Ghanbari Motlagh, Ghazaleh Hajivalizadeh, Zahra Hoseini Tavassol, Afshin Ostovar, Moloud Payab

**Affiliations:** ^1^ Osteoporosis Research Center, Endocrinology and Metabolism Clinical Sciences Institute Tehran University of Medical Sciences Tehran Iran; ^2^ Prevention of Metabolic Disorders Research Center, Research Institute for Metabolic and Obesity Disorders, Research Institute for Endocrine Sciences Shahid Beheshti University of Medical Sciences Tehran Iran; ^3^ Department of Epidemiology and Biostatistics, School of Public Health Tehran University of Medical Sciences Tehran Iran; ^4^ National Institute for Health Research (NIHR) Tehran University of Medical Sciences Tehran Iran; ^5^ Center of Excellence for Global Health, School of Public Health Tehran University of Medical Sciences Tehran Iran; ^6^ Shahid Beheshti University of Medical Science Tehran Iran; ^7^ Obesity and Eating Habits Research Center, Endocrinology and Metabolism Molecular ‐Cellular Sciences Institute Tehran University of Medical Sciences Tehran Iran; ^8^ Endocrinology and Metabolism Research Center, Endocrinology and Metabolism Clinical Sciences Institute Tehran University of Medical Sciences Tehran Iran; ^9^ Non‐Communicable Diseases Research Center, Endocrinology and Metabolism Population Sciences Institute Tehran University of Medical Sciences Tehran Iran

**Keywords:** COVID‐19, health services accessibility, neoplasms, palliative care, telemedicine

## Abstract

**Background and Aims:**

Globally, the emergence of the COVID‐19 pandemic profoundly impacted cancer management. This review discusses the different perspectives on the effect of the pandemic on cancer care in Iran.

**Methods:**

The literature search yielded 38 articles on cancer management in Iran during the COVID‐19 pandemic. This study evaluated multiple aspects of cancer management, including screening, diagnosis, surgical and pharmacological management, palliative care, psychological aspects, telemedicine, cancer research, and guidelines and recommendations during the pandemic.

**Results:**

Studies indicate that cancer patients are at a heightened risk of severe COVID‐19 infections, with a high risk of requiring invasive ventilation, hospitalization in the ICU, and subsequent death. The pandemic significantly affected cancer screenings, in turn leading to a greater number of advanced disease presentations. Furthermore, some surgical and pharmacological treatments were deferred, complicating risk‐benefit assessments resulted in complex and challenging decisions regarding patient care. Cancer patients experienced substantial psychological distress, which was exacerbated by delays in treatment and anxiety about contracting COVID‐19. The commentary highlights the critical importance of adaptive measures, such as telemedicine and home‐based palliative care, to minimize the effects of future health crises on cancer care.

**Conclusion:**

The significant impact of the pandemic on cancer care highlights the critical nature of ensuring that cancer care is a top priority during public health emergencies to achieve equitable access and quality patient care.

## 1 Introduction

In December 2019, an outbreak of unknown pneumonia was reported in Wuhan, China [1]. The following month, the World Health Organization (WHO) announced the pandemic as a “Public Health Emergency of International Concern” on January 31, 2020 [2]. Concerns regarding the possibility of identical events in other regions prompted social, political, and healthcare strategies aimed at mitigating the impact of the crisis. These strategies were significantly influenced by the announcement of a pandemic named “COVID‐19” by the WHO, which was attributed to the severe acute respiratory syndrome coronavirus 2 (SARS‐CoV‐2). Until May 2022, more than 532 million cases of COVID‐19 infection were reported, leading to 6,312,535 deaths [3]. Although the concentrated focus on COVID‐19 was considered essential, experts acknowledged that it adversely affected the management of other health conditions, particularly chronic non‐communicable diseases (NCDs) like cancer, which significantly burden healthcare systems, especially in developed nations [1].

Cancer is a significant challenge for public health, society, and the economy in the 21st century. It contributes to around one in six deaths (16.8%) worldwide and accounts for one in four deaths (22.8%) attributed to NCDs. Additionally, cancer is responsible for 30.3% of all premature NCD‐related deaths among individuals aged 30–69 years. It ranks as one of the top three leading causes of death within this age demographic across 177 out of 183 countries [4]. Also, cancer ranks as the leading disease in terms of economic burden, leading to the redirection of a significant portion of health budgets towards cancer care [5].

Cancer results in increased susceptibility to COVID‐19 infection. According to the WHO, cancer patients have twice the risk of developing COVID‐19 infection as healthy subjects. In case of malignancy alongside anticancer medications (immunotherapy, chemotherapy, and targeted therapy), the risk of life‐threatening infections and events such as the need for invasive ventilation, admission to the ICU, and higher fatality increases up to 3.5‐fold [6, 7]. Furthermore, certain elements triggered during the COVID‐19 pandemic were linked to tumorigenesis and metastasis. The hypoxia, inflammation, increased levels of interleukin 6 and other cytokines, and decreased levels of angiotensin‐converting enzyme 2, characteristic signs of COVID‐19, could potentially trigger metastasis and tumor recurrence [8]. As a result of this mutual effect, community oncologists actively sought methods to sustain their practices while enhancing the standard of treatment for their patients [9]. Hence, special protocols and guidelines released in Iran reflected counter‐measures to substantial challenges regarding cancer management during the pandemic [10].

Annually, more than 131,000 new cases of cancer are reported in Iran. This incidence is expected to rise by 40% by 2025. However, research indicates that 50% of cancers can be prevented. Hence, developing a strategic plan is essential. This underscores the critical role of national cancer control programs, which must offer effective, practical, and affordable services that can be appropriately implemented within a geographic region of a country. Hence, Iran implemented the National Cancer Control Program (IrNCCP) as a comprehensive cancer management model. This program has successfully reached some of its goals within the allocated period [5, 11].

Although cancer programs improved cancer management, as reported, 46% of countries experienced disruptions in cancer treatment services during the COVID‐19 pandemic [12]. Cancer care in Iran also confronted numerous challenges and placed clinical decision‐makers at significant risk of mismanagement [13]. The present review aims to investigate different aspects of cancer management and the associated challenges encountered throughout the COVID‐19 pandemic in Iran.

## 2 Methods

A comprehensive literature search was conducted on April 21, 2024, utilizing a combination of electronic databases and manual searches. Electronic databases included Web of Science, Scopus, PubMed, and Iranian scientific databases (SID and Magiran) to ensure a comprehensive search of both international and domestic literature. To ensure a comprehensive search, the search strategy included a combination of Medical Subject Headings (MeSH) terms and free‐text keywords. The following keywords were used:

- Cancer‐related terms: (“Cancer Control” OR “Cancer Plan*” OR “Cancer Program*” OR “Cancer Prevention” OR “Control of Cancer” OR “Cancer policy” OR “Cancer Care” OR “Cancer management” OR “Cancer services” OR “Cancer screening” OR “Cancer surveillance” OR “Cancer action plans” OR “Cancer task force” OR “Cancer strateg*” OR “Cancer inequalit*”)
- COVID‐19 related terms: (“NCOV” OR “2019 NCOV” OR “COVID‐19” OR “SARS‐CoV‐2” OR “sars cov 2” OR “coronavirus” OR “Severe Acute Respiratory Syndrome Coronavirus 2” OR “COVID”)
- Country‐specific term: (Country = Iran OR Title/Abstract = Iran)

In addition to the literature review, a gray literature search was conducted to gather additional relevant literature (including government documents, reports, and policies) related to cancer care in Iran during the COVID‐19 pandemic.

This review included published research articles, reviews, and policy documents written in English or Persian. Conference abstracts were excluded from the review. The literature search, study selection, and article retrieval processes were conducted and reported in accordance with the Preferred Reporting Items for Systematic Reviews and Meta‐Analyses (PRISMA) guidelines. The initial screening of articles was conducted by title and abstract. Subsequently, full‐text articles of potentially relevant studies were retrieved and critically appraised for inclusion in the final review. Data extraction was performed by a single reviewer to ensure consistency.

## 3 Results

**Figure 1:**
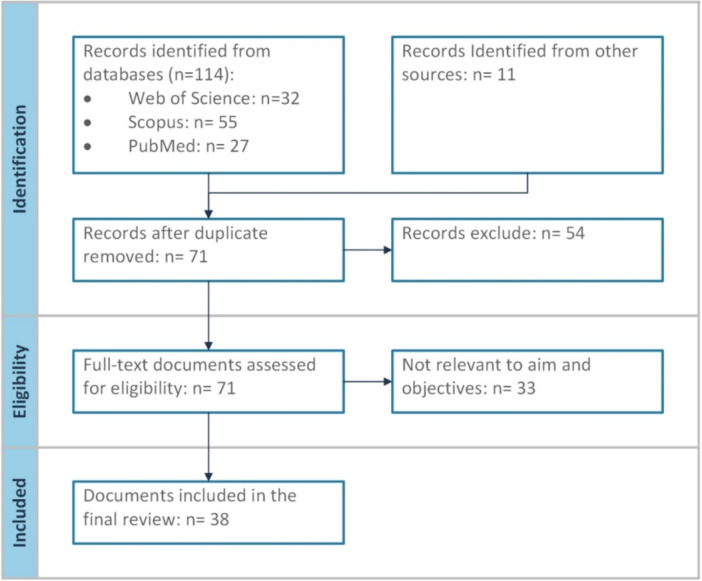
Flow diagram for literature review.

**Figure 2:**
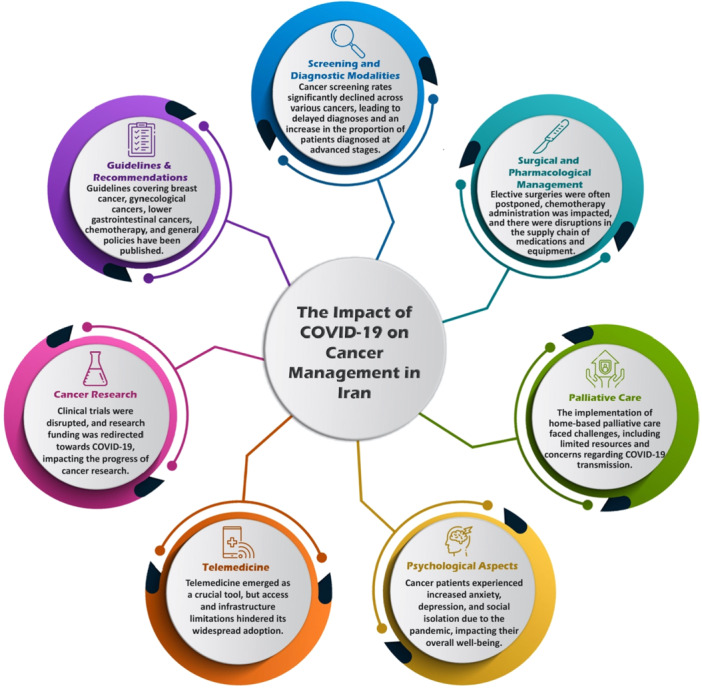
Impact of COVID‐19 on cancer management in Iran.

The search yielded 71 papers, of which 38 met the research objectives and quality criteria after the exclusion of irrelevant studies. Out of the 38 articles, eight were written in Persian, while the others were in English. The selection process for this review is outlined in Figure 1. Studies investigated the impact of the pandemic on various aspects of cancer management, including screening and diagnostic modalities, surgical, pharmacological, and radiotherapeutic management, palliative care, psychological aspects, telemedicine, and cancer research. Furthermore, seven guidelines and recommendations regarding cancer management amid the COVID‐19 pandemic have been published, including the management of breast cancer, gynecological cancers, lower gastrointestinal cancers, chemotherapy, and general policies. Figure 2 provides a visual overview of the key challenges faced by cancer patients and the healthcare system in Iran during the COVID‐19 pandemic.

### 3.1 Screening and Diagnostic Modalities

The COVID‐19 pandemic significantly affected cancer screening and diagnostic processes in Iran, as reported by several studies. These impacts included reductions in screening rates, delays in diagnoses, and, additionally, in breast cancer and head and neck cancer cases, increased prevalence of advanced‐stage cancer presentations was observed [10, 14, 15].

Regarding colorectal cancer, one study found a 12.16% decrease in the screening rate with fecal immunochemical tests (FIT) during the 2 years following the onset of the pandemic compared to the preceding 2 years. This decline extended to subsequent diagnostic steps, such as fewer positive FIT results, reduced patient referrals to the Research Institute of Gastroenterology and Liver Diseases Center, and decreased endoscopic evaluations. Consequently, there was a notable reduction in the detection of sessile serrated polyps, high‐risk adenomas, and early‐stage colorectal cancers [16].

Breast cancer screening services also experienced substantial setbacks. According to one study, average screening rates for breast cancer dropped by 70% between February 22, 2020, and May 24, 2021, compared to the same timeframe the previous year. This decline left approximately 70,914 women aged 30–70 without necessary breast cancer screening services, resulting in delayed diagnoses and more advanced disease stages at detection [15].

Similarly, the diagnosis of head and neck cancers was delayed during the pandemic. Research showed that patients were more likely to present with advanced‐stage tumors compared to the pre‐pandemic period. Contributing factors included limited access to healthcare and patient hesitancy to seek medical attention, leading to overlooked early symptoms and significant diagnostic delays [14].

These findings collectively highlight the widespread disruptions in cancer screening and diagnostic services caused by the pandemic. They emphasize the urgent need for resilient strategies to maintain continuity of care during global health crises.

### 3.2 Surgical and Pharmacological Management

Cancer patients in Iran faced significant challenges in surgical and pharmacological treatments during the COVID‐19 pandemic, as evidenced by several studies. These challenges arose from postponed surgeries, modified chemotherapy protocols, and limited healthcare resources.

In Iran, a reduction in breast surgeries alongside the prioritization of essential surgical procedures was implemented due to the disruption caused by the COVID‐19 pandemic. One study reported that elective surgeries, including breast reconstruction procedures, were frequently postponed, while emergency operations such as drainage for breast hematomas, abscesses, and ischemic flaps were prioritized. Postoperative follow‐ups were also shifted to remote platforms to minimize in‐person interactions [17, 18].

Findings from a study regarding laryngeal cancer surgical management in Iran demonstrated that a significant disparity was observed in the number of referred patients for diagnosis and treatment during the COVID‐19 pandemic compared to the pre‐pandemic period. Patients who sought medical attention during the pandemic presented with higher cancer stages, as well as increased tumor volume, in contrast to those who were referred before the pandemic. Hence, it was not feasible to conduct less invasive procedures, necessitating the performance of a total laryngectomy [19].

Chemotherapy practices underwent significant adjustments. A study highlighted that clinicians opted for less intensive regimens and extended intervals between chemotherapy cycles to reduce patient visits to healthcare facilities. While these adaptations minimized the risk of COVID‐19 exposure, they potentially affected treatment outcomes for certain patients [6, 20].

Resource shortages further complicated cancer care. A hospital in Tehran developed a systematic protocol to maintain chemotherapy delivery during the pandemic while ensuring safety measures were in place. A group of clinical oncologists suggested that individuals with cancer who undergo chemotherapy require specific care regarding their immunocompromised condition [21].

Research findings demonstrated that during the pandemic, a transient disruption in the supply chain concerning chemotherapy agents and medical equipment was observed in Iran. Also, obtaining cancer treatment encountered challenges. The pandemic effects on therapeutic aspects were mainly due to the financial allocation of the essential medications for COVID‐19 management. Additionally, there was a reported shortage of healthcare professionals, primarily attributed to the reallocation of personnel to COVID‐19 management and COVID‐19 infections among staff, alongside a scarcity of medications and equipment in Iran [9, 10].

However, a retrospective study in Iran, which evaluated 279 cancer patients over 90 days, demonstrated that treatment administration following protective protocols resulted in no reported infections among the participants [22].

The studies underline the need for flexible and adaptive approaches to surgical and pharmacological cancer care during health emergencies. By strengthening resource allocation and optimizing treatment strategies, healthcare systems can better navigate future crises.

### 3.3 Palliative Care Services

The delivery of palliative care for cancer patients in Iran was severely impacted during the COVID‐19 pandemic, as evidenced by various studies. Restrictions on hospital visits, resource limitations, and fears of virus transmission posed significant challenges to these essential services.

One study explored the implementation of home‐based palliative care during the pandemic, highlighting its potential to reduce hospital visits and provide safe, effective care in patients' homes. Evidence indicates significant advancements in both palliative care and home healthcare services in Iran. By 2023, 704 licensed home healthcare centers operating across 30 provinces were developed in Iran. However, barriers such as inadequate medication supplies, a shortage of trained personnel, and insufficient infrastructure hindered its widespread adoption [23].

Furthermore, another study exploring the views of a group of participants, varying from patients to oncologists, on home‐based palliative care for cancer patients throughout the COVID‐19 pandemic in Iran indicated opinions on some requirements for delivering this facility. Interviews indicated that establishing home‐based palliative care programs requires reducing COVID‐19 transmission to vulnerable cancer patients by following health recommendations, delivering services through telemedicine as much as possible, and providing guidelines for appropriate management [24].

These findings emphasize the importance of integrating home‐based palliative care and telehealth solutions into healthcare systems to ensure uninterrupted care during crises. Addressing policy and infrastructure gaps will be crucial to improving the quality and accessibility of palliative care services.

### 3.4 Psychological Aspects

The COVID‐19 pandemic mentally impacted cancer patients in Iran and increased their susceptibility to psychological issues. It resulted in many challenges for women diagnosed with breast cancer, including an increased prevalence of anxiety, depression, and impaired cognitive function [25].

One study revealed that approximately 25% of patients experienced stress, while 20% reported moderate levels of depression and anxiety, indicating elevated psychological distress levels among cancer patients throughout the pandemic. Based on an investigation conducted during the pandemic, around 22.2% of patients experienced stress, and 43.2% of cancer patients experienced moderate anxiety, while 36.4% reported moderate depression during the pandemic, according to delays in cancer care, including screening and treatment, shortages of medications, and insufficient nursing care reported by patients in Iran [26]. Additionally, psychological issues also resulted from a lack of knowledge regarding mental health care when patients encountered the death of their relatives. Restricted access to psychological consulting centers exacerbated these issues, leaving many patients without adequate support [27].

Caregivers also faced considerable stress, including increased caregiving responsibilities and fears about safeguarding immunocompromised patients [26]. In contrast, a study investigating the anxiety levels of 102 breast cancer patients during the pandemic in Iran indicated that their anxiety levels were low [28]. A study investigated the effectiveness of online hope therapy in alleviating anxiety and enhancing coping skills for breast cancer patients during the pandemic [29].

These studies highlight the critical need for accessible and innovative mental health interventions, such as teletherapy, to support cancer patients and their caregivers during public health emergencies. Expanding psychological services and ensuring their integration into cancer care are essential steps in mitigating the long‐term effects of such crises.

### 3.5 Telemedicine

The expansion of telemedicine during the COVID‐19 pandemic played a critical role in ensuring continued cancer care in Iran, as highlighted by multiple studies. These studies emphasize how digital health technologies helped mitigate disruptions in healthcare services while also revealing existing challenges in their implementation.

Studies highlighted that telemedicine facilitated remote consultations and reduced the need for in‐person visits, allowing clinicians to monitor patients safely and efficiently. However, only a limited number of facilities in Iran possess substantial telecommunications structures and extensive hotline capabilities, enabling them to effectively implement telemedicine, which is usually performed via video conferencing in cancer management [13, 30]. Initially, in Iran, managing appointments via phone, inquiring about infection symptoms, and evaluating the necessity for an in‐person visit before attending visits were generally recommended [20].

The findings suggest that telemedicine can play a transformative role in cancer care, particularly during health crises. However, to maximize its potential, investments in infrastructure and training are needed to address the barriers to widespread adoption.

### 3.6 Cancer Research

The national cancer centers highlighted the significant impact of the COVID‐19 pandemic on international collaboration in Iran [10].

These findings underscore the importance of developing contingency plans and leveraging technology to sustain cancer research during emergencies. Ensuring the resilience of clinical trials is essential for advancing cancer care and treatment outcomes, even in the face of global crises.

## 4 Discussion

The COVID‐19 pandemic had profound and multifaceted impacts on cancer care in Iran, as evidenced by disruptions in screening, diagnosis, and treatment. Delays in cancer screenings and diagnostic services resulted in reduced detection rates for colorectal, breast, and head and neck cancers, often leading to advanced‐stage diagnoses [10, 14, 15, 16]. Additionally, modifications to treatment protocols and significant postponements in surgeries further complicated patient management [9, 10, 13, 31]. The pandemic also highlighted the psychosocial toll on cancer patients, exacerbating anxiety, depression, and stress [25, 26]. Despite these challenges, adaptive measures such as telemedicine and home‐based palliative care emerged as vital strategies to mitigate the effects of the crisis on cancer care delivery [10, 13, 24]. Furthermore, guidelines addressing cancer management during the COVID‐19 pandemic have been published [6, 17, 20, 21, 31, 32, 33]

On a global scale, healthcare systems faced challenges in sustaining cancer care throughout the COVID‐19 pandemic, as well [34]. The pandemic had a substantial impact on the management of cancer patients in primary care settings in Iran, including all stages, from diagnosis to treatment and follow‐up [15]. Consistent with the results of our study demonstrating disruption in the screening and diagnostic modalities, the COVID‐19 pandemic deeply affected cancer screening, diagnosis, and treatment globally [35]. However, the aforementioned implemented adaptive strategies, such as leveraging telemedicine and restructuring treatment schedules, mirror those seen internationally [9, 36, 37]. This underscores the universality of these solutions in crisis management.

During strenuous eras such as COVID‐19, due to the time‐sensitive nature of cancer therapy, the healthcare system was compelled to prioritize digital and instantaneous techniques that minimize patient attendance. Previously, telemedicine has been well‐received by both patients and physicians. Implementing this strategy during the pandemic was suggested to enable efficient management of patients' conditions without unnecessary delays and play a critical role in pandemic preparedness and response. The emergence of the COVID‐19 pandemic led to a substantial expansion of electronic health services, greatly facilitating healthcare delivery to patients and communication with specialists. The overall consensus among the public was overwhelmingly positive regarding these services [36, 38, 39]. Based on the results of our study, virtual consultations and telemedicine were utilized in Iran to improve patient care and address disruptions in cancer management during the pandemic, although their implementation faced limitations due to inadequate infrastructure [10]. Addressing these technological and systemic gaps could enhance the resilience of cancer care systems in future crises.

The national cancer centers highlighted the significant impact of the COVID‐19 pandemic on international collaboration in Iran [10]. Generally, the pandemic is expected to negatively affect cancer research in the clinical and basic sciences. Since conducting clinical trials typically necessitates multiple patient visits to healthcare and research facilities, along with numerous interactions among patients, clinicians, and research coordinators [30]. This highlights the need for contingency planning and increased funding to sustain clinical trials and research activities during emergencies. Strengthening collaboration among researchers, healthcare providers, and policymakers is critical to ensuring uninterrupted progress in cancer research.

The strengths of this study lie in its comprehensive investigation of the impact of the pandemic on various aspects of cancer care in Iran and its focus on identifying adaptive strategies. However, it is limited by several limitations, particularly the limited number of investigations on the impact of the COVID‐19 pandemic on cancer management in Iran. As a result, certain aspects of this impact were not thoroughly evaluated. Additionally, some of the included studies were not original research; they comprised editorials that reflected the authors' opinions on the global and national effects of the pandemic on cancer management. Future research should explore the long‐term effects of these disruptions on patient outcomes and the effectiveness of implemented strategies. Addressing these gaps will provide valuable insights for enhancing cancer care resilience in the face of future public health emergencies.

## 5 Conclusion

The COVID‐19 pandemic exposed critical vulnerabilities in cancer care systems, particularly in countries like Iran, where healthcare resources and infrastructure were already under strain. This study highlights the substantial disruptions in cancer screening, diagnosis, surgical and pharmacological treatments, and palliative care services, which collectively led to delays in diagnoses, advanced‐stage presentations, and compromised patient outcomes. Moreover, the psychosocial toll on patients and caregivers further compounded these challenges.

Despite these obstacles, the pandemic also catalyzed the adoption of adaptive strategies, such as telemedicine and home‐based care, which played pivotal roles in maintaining some degree of continuity in cancer care. However, limitations in technological infrastructure and systemic preparedness restricted their widespread implementation. These findings underscore the need for robust, crisis‐resilient healthcare policies prioritizing the continuity of cancer care even during emergencies.

Investments in digital health infrastructure, resource allocation, and collaborative policymaking are essential to build a more resilient cancer care system. Addressing systemic gaps and ensuring equitable access to cancer care services must remain central to future healthcare strategies. The lessons learned from the COVID‐19 pandemic provide a unique opportunity to fortify cancer care systems and improve patient outcomes during future public health crises.

## Author Contributions

**Sepideh Hajivalizadeh:** investigation, conceptualization, writing – original draft, writing – review and editing. **Mohammad Javad Mansourzadeh:** investigation, visualization, writing – original draft, writing – review and editing. **Arash Ghazbani:** methodology, writing – original draft, writing – review and editing. **Elham Ehsani:** writing – original draft, writing – review and editing. **Ali Ghanbari Motlagh:** writing – original draft, writing – review and editing. **Ghazaleh Hajivalizadeh:** writing – original draft, writing – review and editing. **Zahra Hoseini Tavassol:** writing – original draft, writing – review and editing. **Afshin Ostovar:** methodology, conceptualization, supervision, writing – review and editing. **Moloud Payab:** conceptualization, supervision, writing – review and editing. All authors have read and approved the final version of the manuscript corresponding authors had full access to all of the data in this study and take complete responsibility for the integrity of the data and the accuracy of the data analysis.

## Ethics Statement

The authors have nothing to report.

## Consent for Publication

All authors edited and approved the final version of this manuscript for submission.

## Conflicts of Interest

All authors declare that they do not have any potential or perceived competing interests. The funding sources had no role in the study design; collection, analysis, or interpretation of the data; writing of the manuscript; or the decision to submit the article for publication.

## Transparency Statement

The lead authors, Afshin Ostovar and Moloud Payab, affirm that this manuscript is an honest, accurate, and transparent account of the study being reported; that no important aspects of the study have been omitted; and that any discrepancies from the study as planned (and, if relevant, registered) have been explained.

## Data Availability

This study is a review based exclusively on previously published literature. No new datasets were generated or analyzed in this study. All data supporting the findings of this review are available within the cited articles included in the reference list.
